# Predictive Maintenance for Pump Systems and Thermal Power Plants: State-of-the-Art Review, Trends and Challenges

**DOI:** 10.3390/s20082425

**Published:** 2020-04-24

**Authors:** Jonas Fausing Olesen, Hamid Reza Shaker

**Affiliations:** 1 Center for Energy Informatics, University of Southern Denmark, 5230 Odense, Denmark; jonol@orsted.dk; 2 Ørsted, Markets & Bioenergy, Asset Risk Management, Kraftværksvej 53, 7000 Fredericia, Denmark

**Keywords:** machine learning, predictive maintenance, remaining useful lifetime, state of the art review

## Abstract

Thermal power plants are an important asset in the current energy infrastructure, delivering ancillary services, power, and heat to their respective consumers. Faults on critical components, such as large pumping systems, can lead to material damage and opportunity losses. Pumps plays an essential role in various industries and as such clever maintenance can ensure cost reductions and high availability. Prognostics and Health Management, PHM, is the study utilizing data to estimate the current and future conditions of a system. Within the field of PHM, Predictive Maintenance, PdM, has been gaining increased attention. Data-driven models can be built to estimate the remaining-useful-lifetime of complex systems that would be difficult to identify by man. With the increased attention that the Predictive Maintenance field is receiving, review papers become increasingly important to understand what research has been conducted and what challenges need to be addressed. This paper does so by initially conceptualising the PdM field. A structured overview of literature in regard to application within PdM is presented, before delving into the domain of thermal power plants and pump systems. Finally, related challenges and trends will be outlined. This paper finds that a large number of experimental data-driven models have been successfully deployed, but the PdM field would benefit from more industrial case studies. Furthermore, investigations into the scale-ability of models would benefit industries that are looking into large-scale implementations. Here, examining a method for automatic maintenance of the developed model will be of interest. This paper can be used to understand the PdM field as a broad concept but does also provide a niche understanding of the domain in focus.

## 1. Introduction

Production facilities are built with increasing complexity to satisfy the increase in demand for quality products and availability. To deliver on this, manufacturers need to understand the current state of their assets to avoid unnecessary downtime [1,2]. The maintenance procedure can be compared to the health care procedures conducted on man. If conducted efficiently, the system will be operating under fewer restrictions and prove to have higher performance. This means cost reductions, resource-saving, increased life expectancy, and reduced downtime. On the other hand, poor maintenance management can result in a reduction in safety, reputation, and quality of product delivered [3]. Hence, the question becomes *when* maintenance should be deployed. Traditionally maintenance had been done either reactive or preventive [4]. The reactive maintenance approach allows components and machinery to run till failure. The consequence is long downtime and thus a market opportunity loss. The preventive maintenance approach deploys maintenance based on a time or cycle metric. This is seen in cars going x kilometers before going for service or jet engines doing x cycles. This significantly reduces downtime, as the metric is decided based on expert knowledge. The downside is that asset operators do not know whether the maintenance is deployed efficiently [2,4]. If the engine could go longer without maintenance a result would be poor resource management. To answer the *when*, a just-in-time maintenance approach would be optimal [5].

### 1.1. The Role of Power Plants and the Need for Pumps

Combined heat and power plants, CHP, are an important asset in current energy infrastructures, as they can provide power, grid stability, and heating simultaneously. With increasing amounts of intermittent renewable capacity being installed both power grids and CHP operators are pressured. Power grids face larger instability, while CHP operators are pressured by low electricity prices due to cheap renewable energy [6,7]. Hence, power grid operators depend on the availability and quality of the product provided by the CHPs, while CHP units strive to stay competitive. Though depending on the energy market framework, the CHPs tend to be competing with each other and thus the plant with higher efficiency or flexibility tend to be activated. This means that asset managers at all time need to know, which plants require maintenance to reduce downtime at high electricity price hours. If a critical component fails at a bad time, it can cause severe damage and represent a significant loss. Hence, knowing when to conduct maintenance to reduce downtime is of great importance. CHPs larger than 300 MW can have more than 100 pumps installed, varying in size and type, which speaks to the complexity of the system [8]. A feedwater pump failing results in a large capacity reduction or full shut-down depending on the system setup, which in turn presents an opportunity loss and safety issue for asset operators. Avoiding this is desirable. Pumps are utilised in various fields and comes in various forms, but tend to be critical components for operation. Examples are seen in seawage industries and aviation [9,10].

### 1.2. The Field of Prognostics and Health Management

Continuous improvements in information and communication technology, ICT, have increased data accessibility and in turn created a basis for Industry 4.0. This allows for increased automation and data exchange, through increased deployment of Internet-of-Things, IoT, sensors [11,12]. The field dealing with these complex systems and benefits from the development in technology is the study of Prognostics and Health Management, PHM. PHM present tools to understand complex systems, develop health indicators, and predict future complications. PHM has allowed for conducting Condition-based Maintenance and Predictive Maintenance, PdM [1,2].

**Figure 1:**
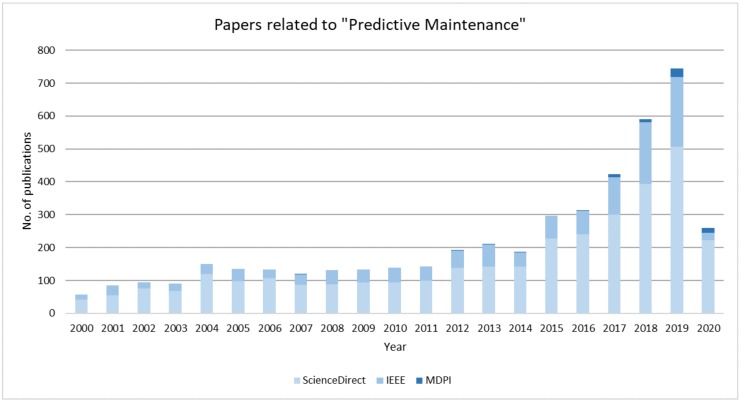
The published papers on predictive maintenance from 2000 to 2020 on MDPI, ScienceDirect and IEEE.

The prognostics within maintenance have been studied to a great degree within literature and has been found to be gaining interest, as new papers are being released at a rapid pace. Figure 1 depicts the trend on three recognised databases considering PdM. This emphasizes the importance of literature reviews. The focal point of literature reviews is to recap what has been investigated, while also determining what challenges and unmet needs require examination.

Prior art conducting reviews within the field of PHM can be found in Table 1. It was found that literature reviews covered a great area of interests, such as algorithms, applications, decision-making, implementation, etc. The thing they have in common is that they are scoped broadly to cover various industries and fields. Thus, the purpose and novelty of this paper is the focus it provides on specific topic. Carvalho et al. [13] presents a structured review of new literature presented in the years after 2014 and does so in a general manner, so the reader can get a general understanding of what moves within the field. Merkt et al. [4] studies various maintenance approaches and the different applications that PdM might have. This is done by studying the full cycle of developing a successful model. Lee et al. [2] outlines a framework for considering to what extent a company will be gaining value from various PdM approaches. It more specifically dives into rotary components and their common failure types, before presenting a set of case studies. This paper will provide an understanding of newly published art within the PdM field and then delve into PdM within pumping systems and CHPs. This allows for a niche understanding of the differences between the general trends and the specific studied field. This approach can be applied to other fields or industries to evaluate gaps.

This paper investigates the literature within the field of PdM, creating a structured overview of current applications. A deep dive into applications found on pumping systems will be provided, before elaborating on how this is or could be applied to a CHP setting. The findings on pumping systems can be extended to other fields. The paper will among other outline the current trends and challenges. One of the focus points will be the scale-ability of the models as systems tend to rely on more than one pumping system. The paper is structured as follows; Section 2 introduces the concept of PdM and puts it into context for the reader. Furthermore, shortly introduce how models can be developed and what is required of the model owner. Following this, state-of-the-art applications will be presented alongside the advantages and limitations of the algorithms considered. Section 3 will then delve into the applications found within pumping systems and CHPs, this is done by presenting an initial overview of common faults within pumps. Section 4 will be presenting the challenges that PdM currently faces. Section 5 will present the future trends found for PdM and what unmet needs that require investigation. Section 6 finalises the paper with a conclusion on the review.

## 2. Predictive Maintenance

### 2.1. Defining Predictive Maintenance

To better grasp PdM, the authors have decided to initially define the concept. According to Oxford Learner’s Dictionaries the adjective *predictive* is formally defined as: “Connected with the ability to show what will happen in the future” and *maintenance* is defined as: “The act of keeping something in good condition by checking or repairing it regularly” [17]. Hence, combining the two will allow for the following definition of PdM; “the act and ability to keep something in a good condition by understanding what happens in the future”.

The following definition of PdM is provided by Mobley [18]; “Predictive Maintenance is a philosophy or attitude that, simply stated, uses the actual operating condition of plant equipment and systems to optimize total plant operation. A comprehensive predictive maintenance management program uses the most cost-effective tools (e.g., vibration monitoring, thermography, tribology) to obtain the actual operating condition of critical plant systems and based on this actual data schedules all maintenance activities on an as-needed basis.” Therefore, PdM strives to identify trends, anomalies, degradation at an early stage, so that sufficient counter measurements can be deployed. The term PdM and CDM has found different use in various papers. Some define it to differ, while others use it interchangeably. This paper will be using the later, as the field of CDM has somewhat developed into a PDM-CDM field. The PDM-CDM considers a broader area of both fault detection, diagnostic, and prognostics.

### 2.2. Methods of the Predictive Maintenance Field

**Figure 2:**
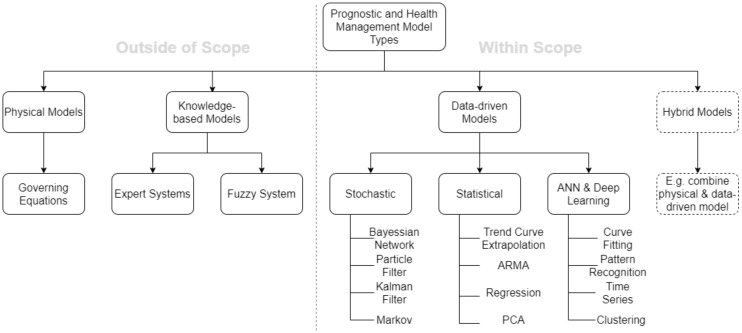
A representation of model types that can be developed and utilised within the PdM field.

Several methods of doing PdM exists, where each approach has a set of pros and cons. It is commonplace to establish system models as they automate the prognostics and can continuously monitor complex system effectively, furthermore, provide indicators of potential risks. Figure 2 presents an overview of common model types utilised. The physical model approach has in current literature been referred to as a digital twin [19,20,21]. The physical laws and formulas governing the system is utilised to establish a model that represents the machinery with great detail. The benefit of such models are the accuracy and knowledge of the system that it provides. White-box models allow for the identification of faults, as the full system is described. This type of modelling have been found to have high accuracy. The weakness of digital twins is the time they consume before being established [21]. Knowledge-based models enable the use of domain expert knowledge and deploy it in models, this can be beneficial as it can assist the prediction process [22]. Data-driven models mainly apply machine learning, soft computing, and statistical theory to establish a model that takes in historical operational or condition data. Though, in many cases not as accurate as physical models, data-driven models can achieve high accuracy. Relative to the amount of time it takes to establish a digital twin, a data-driven model can be developed fast. The weakness of data-driven models is their need for a large amount of high-quality data [21]. If the data is faulty, it will be reflected in the model and the accuracy will deteriorate. Pre-processing methods exist to prepare data and up the quality. Physical and knowledge-based models are as an initial stance not considered to be within the scope of the paper, but is still included to the extent that some papers propose a hybrid approach. Hybrid models combine two approaches and hence utilises the strength of each method to overcome the weaknesses of the other [23]. The focal point of this paper is within data-driven models, but the authors saw the need to mention other applicable approaches and recommends the interested reader to consider the following book by Lughofer and Sayed-Mouchaweh [24]. Lughofer and Sayed-Mouchaweh [24] presents the concepts and applications of the various PdM approaches and introduces a set of case studies.

### 2.3. Framework for Developing a Machine Learning Model

**Figure 3:**
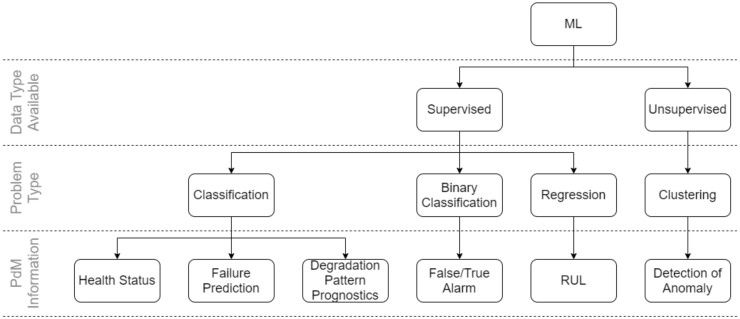
An overview of various methods that can be chosen depending on what information is desired and to what extent data is available.

This section will give an overview of how literature tend to approach creating a data-driven model. First, understanding what type of data you have at your disposition will allow for narrowing down what type of model will be suitable and what information you can achieve. Figure 3 gives an overview of what type of approaches exists. Initially identify whether the data is labelled. If labelled, a broad set of options are available within the realm of supervised methods. If unlabelled, the typical problem type will be that of unsupervised, which allows for clustering. If a different type of information is desired, then manual labelling is possible or it would require a change to the current management of data, by creating a metric that identifies conditional data of the monitored system.

**Figure 4:**
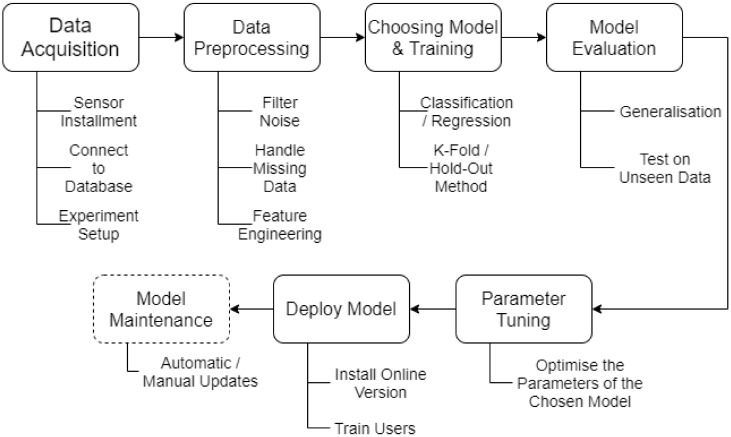
An overview of the process for developing a successful machine learning model, be it in a PdM setting or another.

**Figure 5:**
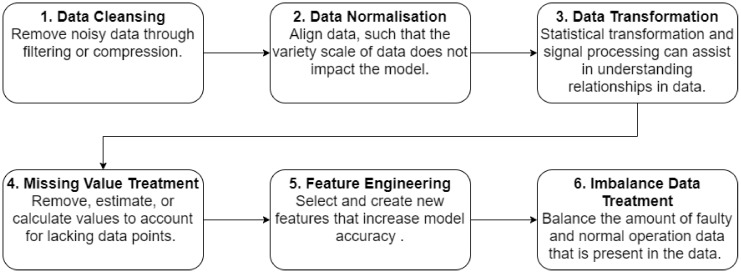
A suggested Taxonomy for Data Preprocessing. The exact order does not necessarily matter and each step should be considered whether necessary for the application at hand.

It seems that a consensus on how a model should be build have somewhat been reached within literature. Though, the order of the steps might be defined or prioritised differently, the same steps tend to be included. Figure 4 depicts an overview of the common steps taken to develop a machine learning model. *Data Acquisition* refers to the gathering of data. With the deployment of communication technology, sensors have been a key source of data, sending and storing data in databases. *Data Preprocessing* is the steps taken to prepare the data. A taxonomy, suggested by Cernuda [25], prepares six steps to consider and is displayed in Figure 5. The exact order depends on the data being worked on. *Choosing Model & Training* considers the amount of available models and the task of training it. In some literature, these are split into two steps, which the authors also consider a possibility. The amount of models available has rapidly increased and gaining an overview can be a challenge, thus finding an appropriate model requires testing. In the paper by Lee et al. [2], a table over a set of models and tools have been proposed alongside their corresponding strengths and weaknesses. A paper by Fernandez-Delgado et al. [26] present an extensive study on various machine learning techniques. Training can often be used as a basis for choosing a model, due to the training commonly giving an indication of the model performance. Methods commonly deployed in literature to evaluate training performance is the k-fold method and hold-out method. The k-fold method splits the data such that a k*th* of the data is used to predict upon and the rest for training, this being iterated k times. The hold-out method is often recommended for larger data sets. This method takes out a percentage of the data to be predicted upon, where the rest is used for training. *Model Evaluation* considers the performance of the model on an unseen test data set. This allows the developer to determine how the model potentially will perform prior to being deployed. Analysing whether the model accuracy found in training aligns with testing. *Parameter Tuning* allows for optimising the accuracy of a model. Machine learning models operate with different parameters to allow for flexibility and to increase or reduce bias. The last two steps consider deploying and maintaining the model. Deploying the system can relate to installation or integration of software, while it can also refer to soft values, such as Change Management for the customer to adopt the new tool and use it for decision-making. Model maintenance considers that systems often are dynamic, where a common assumption on a model is that the system is static [27].

Another common acknowledgment is that the individual case study requires a different approach. Despite this, efforts have been invested in developing a structured approach to create and streamline model construction. The authors recommend the interested reader to consider Lee et al. [2] for an overview of what considerations should be done before determining whether predictive maintenance is something that should be deployed, furthermore on how to approach it effectively. The above discussed problem was popularised as the *No Free Lunch Theorem* by Wolpert and Macready [28] for optimisation. This refers to that no single algorithm is superior to the other, as they each serve a purpose and is case dependent.

### 2.4. Overview of Methods

This section will be introducing relevant literature on successful applications of predictive maintenance. The concept of each highlighted ML method will be introduced shortly before relevant literature will be presented. Machine learning methods tend to be grouped by their “way of functioning”, e.g., tree-based models, such as decision trees, or neural networks with their various architectures. The authors have tried to take this *grouping-by-similarity* approach, but the authors are aware that some might fall into two categories or not fall into any category. An overview of relevant literature categorised can be seen in Table 2. The reader should be aware that papers can have applied several types of methods for either testing or comparison, this allows for the paper to be mentioned several times within the list. Listing the papers show a fair distribution of papers across all categories, but a preference towards research within ANN & DL can be seen as it has been increasing in popularity. Furthermore, it is found that a significant amount of papers are presented in a experimental setting and that approximately 30% of the studied literature utilised vibration data to determine the health status of a system. Though, the majority of prior art is presented in a experimental setting, the applications does span over a large area, which shows the flexibility of the PdM field.

#### 2.4.1. Stochastic Algorithms

Stochastic data-driven models within prognostics are often considered within the Bayesian category. Rather than giving a single estimated output on the current system health, it gives a probability distribution of possible likely options [22]. In this way, the Bayesian method can present the current state of the system, but can also evaluate future trends before a given threshold. Stochastic algorithms are mainly used within degradation models and the most common Bayesian network algorithms are Particle Filters, Kalman Filters, and hidden Markov models [29,30].

Degradation can be difficult to determine, especially, when the progress status is hidden. This means that a degradation can to some extent seem linear, but in reality contain non-linear elements. Furthermore, data can be noisy to the extent that it greatly influences the predicted RUL and hence reduces accuracy. Son et al. [48] delves into this issue. A method is proposed to utilise condition monitored data to predict the RUL with an acceptable error. The RUL is estimated by deploying a model based on a constrained KF model. To do so, a set of inequality restraints are set up to achieve the desired accuracy. To verify the method, it is tested on a case study of an automotive lead-acid battery. Qiu et al. [30] also studies a battery, but looks at both state of charge, state of health, and RUL. To improve the accuracy of the prediction, BS-SRCKF is deployed for the state of charge, which in turn is combined with MHKF and an EKF to do a joint estimate of the state of charge and state of health. The state of health values are then utilised in a particle filter that relies on an improved cuckoo search to determine the RUL.

In an alternative approach, Xue et al. [62] deploys an AUKF alongside genetic algorithm that optimises the parameters for a SVR model. The AUKF serves the purposes of calculating the process noise covariance and observation noise covariance, this is done continuously over the time series. The method is verified on a battery data set provided by NASA.

In the study proposed by Ruiz-Sarmiento et al. [51], machinery degradation in the hot rolling process is studied. This is done by applying a Bayesian Filter. This utilises expert knowledge along side historical data to increase the accuracy of the predictions. The filter deployed is called a Discrete Bayes Filter. The findings of the paper result that this hybrid model relying on expert knowledge and machine learning theory increases the overall accuracy than they could individually.

#### 2.4.2. Statistical Algorithms

Statistical data-driven models within prognostics are relatively simple trend extrapolation. They rely on condition monitoring data to create a trend curve from a single dimension time series that then reflects the degradation of health for the asset. Where stochastic algorithms give a probability distribution as an output, the statistical algorithms give a single specified output. A common algorithm is ARIMA, but it is also possible to apply regression techniques [22].

SVM sees its use in both classification and regression problems. In the paper by Yan et al. [55], the RUL of bearings is estimated based on a classification. This is done by splitting the degradation into 5 categories. The method requires a dimensionless input. This is achieved by calculating the RMS for the vibration data. To evaluate the model, data from a public data set is utilised. The data set considered are from IMS and PRONOSTIA. The model performs equally to better than other models proposed to estimate RUL.

In a paper by Susto et al. [61], SVM is utilised to classify whether the machinery is in need of maintenance. The machinery in consideration is that of an ion-implanter tool. The base case is the current PvM scheme. The study finds that the PdM approach can perform equally to better than the presented PvM scheme. In an approach to detect faults early, LS-SVM Regression is applied to a Vertical Form Fill and Seal. The paper by Langone et al. [57] initially applies an unsupervised clustering algorithm, KSC, to initially identify anomalies within the data set, before applying the LS-SVM regression for prognostics. The model successfully detects when the dirt is building up within the machinery.

In the study by Li et al. [56], LS-SVM is applied in an alternative manner to a distillation process. The data set is based on a moving-window and hence applies just-in-time maintenance. Here the work focuses on creating an online soft sensor tool that allows for monitoring the distillation process. A comparison between applying LS-SVM as a static and dynamic version is done for presenting the results. The static LS-SVM arrives at a $R^{2}$ of 0.995 with a RSME 1.7991, where the dynamic version, with the largest moving window, presents a $R^{2}$ of 0.999 and a RMSE of 0.062.

An alternative approach proposed by Zhou et al. [44], considers algorithms across the categories of statistical and ANN. The RUL prediction is conducted on a PEM fuel cell. The prediction is divided into three phases. The first phase deals with non-stationary trends by deploying PAM. The second phase, the order of an ARMA model is estimated and deployed to filter the linear degradation elements in the data. The final phase utilised the leftover non-linear elements to train a TDNN. The paper finds the model to predict RUL with high confidence.

#### 2.4.3. Artificial Neural Network and Deep Neural Network

The idea of ANN stems from the functionality of the human brain. The nervous system is the driver behind everything that a human does and the nervous system consists of neurons. An ANN replicates this by having a set of layers, where each layer consists of neurons. Each layer is connected and can receive and transmit signals, corresponding to the synapse within the nervous system. Each connector has a weight, the neurons have an activation function and a threshold value. These are the parameters considered within ANN. As technology has developed and ANN has taken on increasingly complex tasks, a new term has been given to more complex ANN, which is called Deep Neural Network. The most distinct difference between the two terms is the complexity, referring to the architecture of the networks and the number of layers that it may contain [22,63,64].

In a paper presented by Silva and Capretz [38], two fans are studied by applying CNN. CNN is typically known for being applied to image recognition, but is in an alternative approach applied to fault detection and predictive maintenance. The features used are divided into electrical, mechanical, and temporal. Initially, features are one-dimensional, hence a data transformation is required. The paper is inspired by Wang and Oates [65] and Chen et al. [66] to try GAF and Moving Average Mapping, respectively, and converts the data into two-dimensions. The intent is to allow for better knowledge on the relationship between features. CNN is applied and finds an accuracy of 95% and 98% for the two fans. The proposed method reports that it outperforms traditional methods such as RF, SVM, and MLP.

In a study proposed by Markiewicz [42], predictive maintenance of induction motors is considered. The paper touches upon the issues of data gathering and storage, as it is an energy intensive process. Hence, Markiewicz [42] presents a solution where predictive maintenance is done locally on a set of ultra-low power wireless sensors. This is possible with a reduced computational complexity from applying a compressed RNN. For their cell, LSTM is applied as they argue that it does not suffer from a vanishing or exploding gradient problem [63]. By applying a compressed RNN algorithm they found an accuracy of approximately 92% and that this was comparable or at a better performance than other algorithms e.g., KNN. This approach moreover benefited from the lowered energy consumption.

Zhang et al. [67] investigates a degradation model on bearing performance by applying LSTM RNN. The model builds on vibration data, but also introduces an alternative indicator, namely “waveform entropy”. Entropy can be for a discrete instance of random vector X be defined as follows:

(1) $$E=\frac{1}{M}\sum_{i=1}^{M}p\left(x_{i}\right)\cdot ln\left(p\left(x_{i}\right)\right)$$

where the *E* is the entropy, *M* is the number of elements in X, $x_{i}$ is a single element of X, and $p(x_{i})$ is the probability mass function. The paper sets out to develop a dimensionless indicator. This is due to e.g., load and speed not only resembling the health of a system, but also reflects the current operating condition. Inspired by Ali et al. [45], a waveform entropy can be calculated:

(2) $$WFE_{t}=\frac{1}{M}\sum_{i=0}^{M-1}W_{t-i}\cdot log\left(W_{t-i}\right)$$

Here WFE is the waveform entropy, $W_{t}$ is the waveform factor at time instance *t*, and *M* is the length of the sliding window. This method benefit from not requiring signal decomposition, such as other time-frequent domain features. WFE is a local mean of logarithmic vibration energy. Hence, a strong representation of the state of the vibration levels. The paper presents three steps for predictive maintenance. Fault detection, fault classification, and a degradation model. The WFE supports this and gives great results.

#### 2.4.4. Clustering

Clustering represents a category of unsupervised algorithms, which objective is to find clusters within a data set. Algorithms typically rely on a centroid or hierarchical approach to determine the clustering of data that reflects the shortest distance internally and the largest distance between clusters. Examples of algorithms are k-means, Expectation Maximisation, Hierarchical Clustering.

As clustering is unsupervised, the main purpose of clustering algorithms is to find golden nuggets within the data. In a PdM setting, the main goal of clustering becomes to detect failure and anomalies within data. In a paper by Uhlmann et al. [68], k-means is deployed in a supervised setting to see the performance of detecting faults on a Selective Laser Melting machine tool.

Another approach is using clustering alongside other algorithms. This is done in the work by Cao et al. [69]. Here fuzzy clustering is deployed to determine the severity of a failure, before deploying semantic technologies to investigate the time of the failure. Similarly, Daher et al. [70], applies fuzzy C-means alongside ANFIS to detect the RUL of a distillation column.

### 2.5. Advantages and Limitations

In this section, some of the strengths and weaknesses of the investigated algorithms will be elaborated upon. Considering that the various types differ greatly, each has its purpose and application. Table 3 lists some general advantages and limitations of the three categories. This is not a complete list, as the individual architecture within ANN & DL and algorithm within regression present a set of strengths and weaknesses. This is considered out of scope for this paper. For a look into benchmarking of ML, the reader is recommended to visit Olson et al. [71]. Furthermore, Fernandez-Delgado et al. [26] present a comparison of 179 machine learning algorithms on various data sets. Where Ahmed et al. [72] looks at an empirical comparison of time series forecasting within the machine learning domain. This paper will proceed to discuss some of the specific advantages and limitations found within the literature of the various case studies.

ANN solutions have been gaining speed as new architectures have been discovered [32]. ANN have enabled machine learning to complete more complex tasks that were not previously possible or were difficult by nature. The downside is that it is a black box, meaning low interpretability.

Sampaio et al. [31] found that the MLP architecture worked effectively on non-linear and complex systems, furthermore it had great generalisation. On the downside, the convergence was found to be somewhat slow and that the model had a tendency to overfit. Finally, when the proposed model projected a RUL value near the end of the lifetime, it would slowly move further away.

Zhou et al. [36] claimed that the use of ANN provided great calculation speed. More specifically, the KELM algorithm performed better than other algorithms in generalisation, due to it being able to find a least-square optimal solution. Another advantage being that it can achieve multiple outputs. Though one weakness being that it is heavily dependent on the initial parameters, so if estimated poorly it will be reflected in the model. Similarly, Javed et al. [37] finds that ELM has a fast iterative tuning process for deciding on hidden layer parameters. This is due to the algorithm doing it in a single-step and does not need human intervention. The downside is the random parameters could be initiated poorly.

Markiewicz et al. [42] finds LSTM-RNN to benefit from being relative stable, notably because it does not suffer from the vanishing or exploding gradient problem [73,74]. Zhang et al. [33] states that LSTM-RNN has good performance on sequential data due to the recurrent feedback. Finally, as argued by Nguyen and Medjaher [40], LSTM-RNN benefits from having a long-term memory meaning it can keep important information for later application. This can be especially beneficial for degradation prognostics. Ruiz-Sarmiento et al. [51] decided on utilising a Bayesian approach by applying DAE, this was due to the algorithm being robust against noisy and fluctuating data. They further stated that the reason for not utilising a KF was due to the linearity assumption within the underlying system of KF.

Several papers applied either SVM or SVR algorithms. Here, Li et al. [56] stated that LS-SVM had a good performance on non-linear regression, especially in generalisation. Ruiz-Gonzalez [58] chose a SVM classifier due to the strong generalisation, furthermore, with a small data set the chances of overfitting with SVM were relatively low, as well as having a good computation time. Langone et al. [57] found that LS-SVM benefitted from being able to achieve a global optimum due to least-square presenting itself as convex. Zhou et al. [36] on the other hand states that SVR has a long computational with larger data sets and constrained optimisation issues.

## 3. Applications

The paper has till now studied various state-of-the-art ML approaches on doing PdM and applying algorithms. This general overview is needed when further delving into a specific topic to identify challenges and unmet needs. The focus of this paper lies within pumping systems and with the understanding of the current usage of predictive maintenance, the paper will move further towards what has been accomplished within the field of predictive maintenance on pumps. According to United States International Trade Commission in 2005 [75], 80% of the pumps produced in the world are centrifugal pumps and Karassik and McGuire [76] states that 90% of the pumps used in the chemical industry are centrifugal pumps. Hence, this paper will primarily focus on applications on centrifugal pumps. To finalise this section, understanding what has been conducted on PdM within a power plant setting will also be elaborated upon. As power plants heavily rely on their pumps functioning. Notice that power plants can potentially be using both centrifugal pumps and positive displacement pumps. An article released by Collins and Davis on PowerEngineering [8], gives a great overview of things to consider before investing in one type of pump and states that a power plant of 300 MW will on average have approximately 100 pumps installed.

### 3.1. Common Faults in Pumping Systems

**Figure 6:**
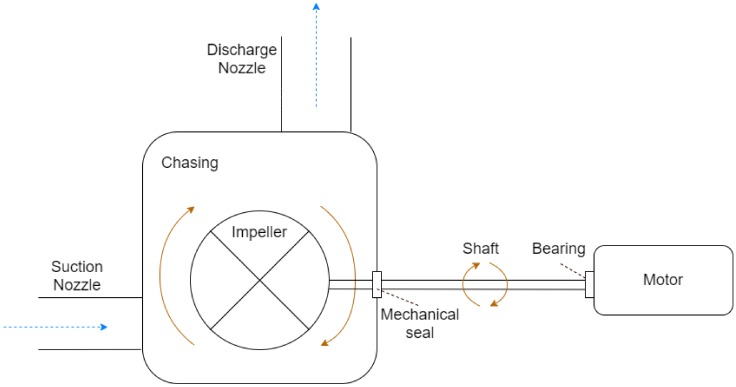
A simple figure of a centrifugal pump. From left to right; water enters the impeller through the suction nozzle, where kinetic energy will be applied to the liquid through turning of the shaft. The chasing keeps the water within the system, while the mechanical seals make sure there is no leakage. The bearing reduces the friction between the moving and stationary parts and is found in several places within a pumping system. The water exits at the discharge nozzle.

A centrifugal pump consists of several rotating and static components that has the task of displacing liquids. Due to the interaction of moving parts and liquids interacting with a solid and static surface, several errors can surface. A thorough investigation by Forbes [9] resulted in a conclusion of dividing the main issues of a centrifugal pump into 13 types of faults. Each of the 13 types of faults could then further be divided into more specific reasons for the fault happening. Table 4 presents an overview of detectable errors with their corresponding data tags that might be of interest. To get a more detailed description of specific issues, the reader is recommended to visit Forbes [9]. The main components of a simple centrifugal pump will consist of an impeller, a suction nozzle, a discharge nozzle, a shaft, a mechanical seal, and several bearings. A more detailed overview can be seen in Figure 6. Due to the nature of the pump, the pump faults can be categorised into three categories. Hydraulic, mechanical, and other failures. The hydraulic failures are caused by the liquid flowing within the piping. The state of the liquid can influence the performance of the pump, but also damage parts of the pump. Mechanical failures refer to the interaction of moving and static parts, this commonly causes wear or other types of fatigue. Finally, other type failures are a category for failures that do not directly fall into the two other groups. The power consumption of a pump can often be an indicator of whether the pump is performing desirably.

### 3.2. Pumps and Thermal Power Plants

Pumps have been around for a long time and seen their application within various industries. Displacing liquid has always been a need for humans to easily transport e.g., water and it is a key part of the modern infrastructure. An example of a common pumping system is seen in CHPs. District heating allows for increased efficiency of power plants by utilising excess heat and deliver heat to costumers. When a critical pump breaks down, it influences the performance of the CHP and hence maintenance becomes of importance to minimise failure and capacity reduction. Table 5 lists algorithms applied to pumps and CHPs found in the literature. It furthermore presents the objective of applying the algorithm and what data was used. Literature on pumps are more commonplace as they serve a general purpose, but it is in most literature limited to fault detection and diagnostic. The literature presented on pumps is not limited to centrifugal pumps as they play a significant role in various systems. Utilising prior art will assist other industries relying on pumps to have an easier time adopt similar systems. The literature found within the CHP domain was very limited, as most of the power plant related material was referring to nuclear power plants, NPP [77,78,79,80]. The authors recognise that the list is not complete and that the list would benefit from a more exhaustive search, but it is representative of current applications.

The difficulty of creating models on pumps and CHPs stems from their flexible nature. NPPs will primarily be operating at high to full capacity at all times. If a NPP is not doing so, it becomes a bad business case for the asset owner. The value of a CHP is, on the other hand, the flexibility that it offers. This is the same for pumps. Depending on the pump it can either freely regulate up and down, or it can have set modes to operate in. Wang et al. [83] presents a model, which initially clusters the operation modes of a centrifugal pump by applying GMM Clustering before estimating a RUL with a PF. Such methods allow for easier fault detection and also eases the process of creating a RUL.

As CHPs are composed of various components, it becomes tricky to create just a single model for the full CHP, but requires a model on component level. Hundi and Shahsavari [89] challenges this, by creating a model which estimates the performance depending on various conditional and time-series data, by applying various regression methods. This later allows for anomaly detection, by presenting a separate model.

The benefit of CHPs being build of various components is that literature from other areas might be applicable. e.g., bearings are commonplace within rotary machinery and state-of-the-art algorithms have been applied to this area [45,49,55,67,91]. Another method of overcoming data reflecting a flexible operation is presented by Moleda et al. [85]. Moleda et al. [85] investigates a feedwater pump on a CHP in Poland. An approach utilising a bag of regression models is suggested. This is done by creating a regression model for each data tag and then calculate the RMSE between the estimated value of the model and the actual value. Though it does not give a RUL estimate, this method allows for detecting drift, anomaly, and outliers.

A trend found within the data sets utilised is that many rely on vibration measurements [81,82,83,84,86,87]. Vibrations are common within pumps, but also generally applied. This is due to vibrations giving a good indication of the current state of health for the given system. Furthermore, given a little pre-processing the degradation curve becomes fairly detectable. In a paper by Tse et al. [87], a slurry pump is studied. Multiple vibration sensor data is extracted and fusioned. Following this a KF is applied to determine the degradation. The proposed method is not reliant on run-to-failure data and can predict the RUL even with one channel failing to provide data.

## 4. Challenges for Predictive Maintenance Applications

A set of challenges were identified, during the literature review. This section will outline some general challenges that PdM faces currently and then some domain-specific challenges concerning pumps and CHP units.

In Table 2 a set of application areas was defined. A large proportion was identified as being experimental [30,32,38,48,50,54]. Meaning that a component was investigated in an experimental setting and hence not necessarily attached to a manufacturer or plant. This shows that even though PdM has come a long way, there still exists a gap between experiments and industrial applications. The field of PdM would benefit from applying algorithms to historic data from industrial processes [92]. This is reasoned that experimental data sets tend to be more fine-tuned, whereas industrial historic data tend to be noisy and have a larger amount of missing data. A sensor might not have been installed for the desired data tag. As seen in Section 3.2, most pump models were developed on vibration data. If vibration data is not available other methods have to be deployed. Furthermore, a component can have multiple sensors as a source for a model, which requires the developer to align and pre-process data [86,87]. Furthermore, industrial data sets, typically, do not have a labelling metric, hence it requires the developer to dive into expert knowledge or company ERP systems to identify faulty data, which in turn can introduce bias. Here, some models present themselves more lucrative, as they do not require a large amount of pre-processing or small amounts of domain knowledge.

Data-driven models tend to make certain assumptions on the system that it reflects. One common assumption is that it is static and does not develop over time [27]. This will in many cases be proven to be wrong with many systems being dynamic. Especially within the field of PdM, as a component is replaced with another, it will require a certain maintenance for each model developed. Several strategies exist to develop models, but currently most of the models are developed offline, before implementation and does not consider maintenance. Here, some methods have been deployed to look at a certain frame or period, e.g., the last year, thus a continuously moving frame. With the deployment of more IoT equipment, new options open up. Some studies within the field of automatic maintenance and continuous improvement have been completed [93].

Another challenge lies in the task of determining an accurate RUL. Depending on the data available different models and methods exist to tackle the problem [44]. In many cases, there is not necessarily run-to-failure data available, but rather a degradation path or rather depend on expert knowledge. The issue then becomes to estimate the RUL from the available data. Some components can have a steady linear degradation at the start of their lifetime, but then suddenly start dropping by the end of it. This is common within e.g., batteries [30,33,54]. Hence, an issue of determining a hidden state process.

In regards to the pump and CHP domain, a challenge was identified concerning the operation. Most experimental data can be measured at a fixed operation, but as CHPs and pumps have variable operation [83], errors may be hidden within the mass of data. Here techniques need to be extended to handle such issues. Another problem is the scale-ability and streamlining of models. As industrial process might have several similar or identical components, it is important to investigate the scale-ability of developed models for them to be used for plug-and-play. This should ease the implementation of PdM into the industrial sector. This is challenged by the *No free lunch theorem*, as a component at one plant might differ slightly from another and then, in turn, prove that another model might be more suitable.

## 5. Future Trends

Following Figure 1, the trend within the field of PdM seems obvious. An increasing interest for research seems to take place. This follows the increase in deployment of ICT enabling data and knowledge sharing. Some companies are already dealing with prior mentioned challenges, such as streamlining and scale-ability of algorithms. e.g., Oracle presents the MSET2 tool that can be deployed for process monitoring [94]. Another company stated that its monitoring tool could significantly reduce maintenance costs [95]. These services will be increasingly common. As tools for easily development and implementation become more common, industrial applications will follow naturally, as it becomes commonplace to utilise the state of the art of PdM to stay competitive.

Another trend that Industry 4.0 brings, is the possibility for automatic maintenance of models [93]. Models will be self-sustainable even with new components being installed, though it will be presenting new issues to deal with as well. Following Moore’s Law, a doubling in computational power can be expected to happen every second year [96]. This will continuously increase the possibilities to solve increasingly complex tasks and open up for various new architectures and algorithms from the ML field.

This paper did not extend to physical and knowledge-based models, only to the point that it was introduced through hybrid models. Though, it seems that increasing amounts of literature is considering combining either data-driven models with physical or knowledge-based models, as this can improve accuracy and trust in the developed model [20,23,36,55]. This can be reasoned that data-driven to a certain degree lack interpretability, where e.g., knowledge-based models would allow for domain experts to influence the model [21]. This might be ideal for the industry moving forward, as it would present them with a larger toolkit to operate within.

## 6. Conclusions

This paper presented a literature review on current state-of-the-art applications within the field of predictive maintenance. The paper initially presented a thorough introduction to what PdM offers and gave a framework on how to prepare and develop PdM models. Here literature was recommended for further reading to deepen the understanding of how model construction could be done. Then literature with applications in various areas were introduced, this allowed for the identification of advantages and limitations of certain algorithms. It further allowed for identifying certain trends within the predictive maintenance field. This was compared to the specific domain of pumps and combined heat and power plants. Here certain gaps were identified, as limited material could be identified within the combined heat and power area. A set of challenges for predictive maintenance were then presented before outlining future trends.

For future work, the authors recommend that a more exhaustive literature review is conducted as there are increasing amounts of literature being published at a rapid pace. General trends could be identified from the studied literature, but the paper would benefit from verification of the exhaustive search. This is especially related to domain-specific areas, as general literature does not seem to be lacking. Furthermore, the literature review should be extended to include literature of other types of models, such as physical and knowledge-based models.

## Figures and Tables

**Table 1:** An overview of a set of review papers in relation to the field of PHM.

Reference	Year	Focus
[13]	2019	Structured PdM literature review of 30 papers and their algorithms. Applications for RF, ANN, SVM, k-means are presented.
[14]	2019	A review of decision making algorithms and their applications.
[15]	2019	Review and proposition of efficient step-wise method for companies to deploy models on historical data and digital twins.
[4]	2019	Review of various maintenance strategies, their application and use-cases with focus on the full cycle of creating PdM models.
[16]	2018	An introduction to CDM with a strong overview over what possibilities it provides.
[2]	2014	A holistic overview of PHM. Present tools for companies to consider to evaluate what degree of maintenance they need. An overview of common fault types on rotary equipment and a list of algorithms with their use-case.

**Table 2:** A list of studied papers categorised by the characteristics of the algorithm applied. The list is an effort on displaying what is moving within the field, but does not reflect all available algorithms.

Category	Algorithm	Area of Application	Type of Machinery	Data	Reference
ANN & DL	MLP	Experimental	Motor	Vibration	[31]
			Aeropropulsion system & truck engine	Nasa & Scania Trucks	[32]
			Battery	Capacity	[33]
		Transportation	Rail	Oil-level, voltage, pressure, temperature	[34]
	ELM	Wind Turbines	Gear	Vibration	[35]
	KELM	Power Production	Hydropower generator	Vibration	[36]
	SW-ELM	Experimental	-	PHM Challenge 2008	[37]
	CNN	Experimental	Aeropropulsion system & truck engine	Nasa & Scania trucks	[32]
		Buildings	Fans	Electrical, mechanical, temporal values	[38]
		Milling	Cutting machinery	Force, feed rate, speed	[39]
	(LSTM)-RNN	Experimental	Aeropropulsion system & truck engine	Nasa & Scania trucks	[32]
			Turbofan engine	Nasa	[40,41]
			Motor bearings	Vibration	[42]
			Battery	Capacity	[43]
	TDNN	Experimental	PEMFC	Voltage	[44]
	SFAM-ANN	Rotational mechanical assets	Bearings	Vibration	[45]
Stochastic	DLM w. BN	Transportation	Aircraft aircondition	Airplane Condition Monitoring System (temperature)	[46]
	Markov	Experimental	-	-	[47]
		Transportation	Rail	-	[29]
	KF	Experimental	Battery	-	[48]
				Voltage, current, capacity	[30]
	SUKF	Experimental	Bearings	Vibration	[49]
	EKF	Experimental	Battery	-	[50]
	PF	Experimental	Battery	-	[50]
				Voltage, current, capacity	[30]
	DBF	Stainless steel industry	Hot rolling process (drums)	Density, temperature, pressure, power, force	[51]
Statistical	LR	Experimental	Industrial radial fans	Vibration, rotational speed, temperature, pressure	[52]
		Milling	Cutting machinery	Vibration	[53]
	RFR	Experimental	Industrial radial fans	Vibration, rotational speed, temperature, pressure	[52]
		Milling	Cutting machinery	Vibration	[53]
	SR	Experimental	Industrial radial fans	Vibration, rotational speed, temperature, pressure	[52]
	ARMA	Experimental	PEMFC	Voltage	[44]
	ARIMA	Experimental	Battery	-	[50]
	RVM	Experimental	Battery	-	[50]
	SVR	Experimental	Battery	Capacity	[54]
	SVM	Experimental	Bearings	Vibration	[55]
	LS-SVM	Refinery	Destillation process	-	[56]
	LS-SVM NAR	Food industry	Vertical form fill and seal machine	Vibration, thermal imaging	[57]
Fault Detection &	SVM	Agro-industry	Harvester	Vibration	[58]
Fault Classification	DT	Transport	Rail	ERP system data, conditional data	[59]
	RF	Transport	Rail	ERP system data, conditional data	[59]
		Milling	Cutting machinery	Speed, feed rate, depth	[60]
	RBF-SVM	ion-Implanter tool	Filament	Current	[61]

**Table 3:** An overview of some of the general advantages and limitations of the algorithms within the given categories [22,63]. As individual algorithms present their own set of strengths and weaknesses the reader is recommended to study the individual algorithm as well.

Category	Advantage	Limitation
ANN	Great w. large dataset	Black box model
	Handles noisy data	Requires a large dataset
	Limited/no need for pre-processing	Computational expensive
	Adaptive nature	
	Deals w. nonlinear & complex tasks	
	Various architectures	
Stochastic	Can deal w. smaller datasets	Require quality pre-processing
	Result w. probability distribution	Require accurate degradation modelling
	Can operate alongside statistical approach	Can struggle w. multidimensional data
	Prior knowledge can be incrementally introduced w. new data to achieve better performance	
	Can deal w. nonlinear tasks	
Statistical	Single precise RUL estimate	Early prediction tend to be inaccurate
	Various types of regression models	Utilise a single dimension for prognostic

**Table 4:** Failure types by category for a centrifugal pump.

Name of Fault	Category	Relevant Data Tag	Description
Cavitation	Hydraulic Failures	Vibration, pump efficiency, noise, pressure, flow rate	Formation of vapour bubbles that collapses and damages the piping system. Can result in fatigue or erosion.
Pressure Pulsation	Hydraulic Failures	Vibration, pressure	Can come from running frequencies of the pump, be ressonance of the system, acoustic behaviour, etc.
Radial Thrust	Hydraulic Failures	Temperature	Thrust directed towards the center of the pump rotor. Typically occurs at low flow rates.
Axial Thrust	Hydraulic Failures	Temperature	Thrust imposed on the shaft in either an inboard or outboard direction. Common consequence is fatigue failure.
Suction and Discharge Recirculation	Hydraulic Failures	Pressure, noise	An unavoidable fault is the recirculation of some water within the impeller, either in suction or discharge of the impeller. Can often be determined by observing pressure pulsations at inlet and outlet.
Bearing Failure	Mechanical Failure	Vibration, temperature, (stress waves/shock pulses)	Can be due to various reasons, such as contamination of bearing oil by water, other liquid, high heat, introduction of solid particles.
Seal Failure	Mechanical Failure	Temperature	Opening of the lapped faces results in solids entering, or solids sticks to the surface and introduces severe wear on the hard face.
Lubrication Failure	Mechanical Failure	Temperature	Excessive heat reduces the lubricating ability and lifetime of the oil.
Excessive Vibrations	Mechanical Failure	Vibration	Stems from unbalanced moving parts, particles of the liquid interacts with the pumping system. A large source of errors should be evaluated to do identification.
Excessive Power Consumption	Other Failure	Voltage, current, impeller speed	A typical indication of the pumping system having a failure somewhere in the system and can have different sources for the error.
Blockage	Other Failure	Flow rate	Clogging of piping system or the impeller can result in the pump stopping to function.

**Table 5:** An overview of relevant literature for the domain of PdM within all types of pumps and CHP units. It is not an exhaustive list, but it can give insight into where more efforts should be focused.

Application	Algorithm	Objective	Data Type	Reference
Pump	GNB, SVM, RF, MLP, KNN	Detect cavitation	Vibration	[81]
	SVM	Detect cavitation & blockage	Vibration	[82]
	GMM Clustering	Detect operation modes	Vibration	[83]
	SOM NN	Fault detection	Vibration	[84]
	Polynomial Regression	Fault detection & diagnostic	Temperature, Pressure, Mass Flow, Current	[85]
	PF	Estimate RUL	Vibration	[83]
	AO-PF	Estimate RUL	oil flow	[10]
	LR, AHSM	Estimate RUL	Flow, vibration	[86]
	KF	Estimate RUL	Vibration	[87]
	LN, MLP, DAE	Estimate RUL	Flow, pressure, stress	[88]
CHP	LR, MLP, SVR, RF	Performance estimation	Temperature, Humidity, Exhaust Vacuum, Full Load Power	[89]
	EE, Autoencoders, IF	Anomaly detection	Temperature, Humidity, Exhaust Vacuum, Full Load Power	[89]
	FL, LR	RUL estimate of turbine	-	[90]

## Abbreviations

The following abbreviations are used in this manuscript:

ML	Machine Learning
RUL	Remaining Useful lifetime
ICT	Information and Communication Technology
PHM	Prognostic and Health Management
PdM	Predictive Maintenance
PvM	Preventive Maintenance
CDM	Condition-based Maintenance
CHP	Combined heat and power plant
NPP	Nuclear Power Plant
ANN	Artificial Neural Network
DL	Deep Learning
MLP	Multilayer Perceptron
ELM	Extreme Learning Machine
KELM	Kernel Extreme Learning Machine
SW-ELM	Summation Wavelet Extreme Learning Machine
CNN	Convolutional Neural Network
LSTM-RNN	Long-Short Term Memory Recurrent Neural Network
TDNN	Time Delay Neural Network
SFAM	Simplified Fuzzy Adaptive Resonance Theory Map (Neural Network)
DLM	Dynamic Linear Model
BN	Bayesian Network
KF	Kalman Filter
SUKF	Switching Unscented Kalman Filter
EKF	Extended Kalman Filter
PF	Particle Filter
DBF	Discrete Bayes Filter
LR	Linear Regression
RFR	Random Forest Regression
SR	Symbolic Regression
ARMA	Autoregressive Moving Average
ARIMA	Autoregressive Integrated Moving Average
RVM	Relevance Vector Machine
SVR	Support-vector Regression
SVM	Support-vector Machine
LS-SVM	Least-squares Support-vector Machine
LS-SVM NAR	Least-squares Support-vector Machine Nonlinear Autoregressive
DT	Decision Tree
RF	Random Forest
RBF-SVM	Radial Basis Support-vector Machine
BS-SRCKF	Backward Smoothing Square Root Cubature Kalman Filter
MHKF	Multiscale Hybrid Kalman Filter
AUKF	Adaptive Unscented Kalman Filter
RMS	Root Mean Square
KSC	Kernel Spectral Clustering
RSME	Root Mean Square Error
R${}^{2}$	Coefficient of Determination
PEMFC	Proton-Exchange Membrane Fuel Cell
GAF	Grammian Angular Field
KNN	k-Nearest Neighbour
ANFIS	Adaptive Neuro-Fuzzy Inference System
GNB	Gaussian Naive Bayes
GMM Clustering	Gaussian Mixture Model
SOM NN	Self Organizing Map Neural Network
AO-PF	Adaptive-Order Particle Filter
AHSMM	Adaptive Hidden Semi-Markov Model
LN	Ladder Network
DAE	Denoising Autoencoder
EE	Elliptic Envelope
IF	Isolation Forest
FL	Fuzzy-Logic
ERP System	Enterprise Resource Planning System
MSET2	Multivariate State Estimation Technique
